# Exploring the Singapore general population’s trust in COVID-19 information from different sources and its association with perceived risk of infection during the pandemic

**DOI:** 10.3389/fpubh.2024.1323543

**Published:** 2024-06-12

**Authors:** Fiona Devi, Bernard Chin Wee Tan, Saleha Shafie, Yun Jue Zhang, Shazana Shahwan, Pratika Satghare, Siow Ann Chong, Mythily Subramaniam

**Affiliations:** Research Division, Institute of Mental Health, Singapore, Singapore

**Keywords:** COVID-19, multi-ethnic, trust, perceived risks of infection, Asia

## Abstract

**Background:**

The degree of public trust in the government’s competence is crucial in preventing the spread of misinformation and reducing psychological distress during a pandemic. The study aimed to (i) explore the trust in COVID-19 information from different sources and trust in the ability of the World Health Organization (WHO), government departments and related institutions in handling the epidemic in Singapore and (ii) its association with perceived risk of infection among Singapore residents.

**Methods:**

A total of 1,129 participants (aged 21 and above) were recruited from a cross-sectional study examining the well-being and resilience of Singapore’s population between May 2020 to June 2021. Trust in COVID-19 information from different sources was measured on a 10-point scale and an ordinal 7-point scale was used for perceived risk of infection. Descriptive statistics and multivariate logistic regression model were conducted.

**Results:**

85.5% reported high trust in COVID-19 information from the government and their ability in handling the pandemic. Participants also reported high trust in COVID-19 information from local public health or infectious disease experts (84.4%) and traditional media (77.2%). Low trust in the ability of government departments and related institutions was associated with higher future (1 month) perceived risk of infection (OR: 5.7, 95% CI 1.02–32.45) and low trust in social media was associated with higher current perceived risk of infection (OR: 2.4, 95% CI 1.09–5.24).

**Discussion:**

The present study provided insight on the level of trust on COVID-19 information from different sources and its associated perceived risks of infection. Future qualitative studies are recommended to facilitate better understanding of public trust and identify strategies for how it can be effectively addressed to support future public health responses.

## Background

The COVID-19 pandemic profoundly impacted societies and economies globally and was constituted as a public health emergency of international concern (1). In essence, governments and stakeholders in public health worldwide have been forced to develop and implement complex healthcare and public health policies to combat COVID-19 following the outbreak (2). During the pandemic, social media platforms were also key for social interactions and community building, especially at the early stage of the pandemic when lockdown orders were implemented (3). Given the multiple sources of information and misinformation across both traditional and social media platforms, the degree of trust in public health authorities defined public perception and reaction to the pandemic (4). Therefore, concern about misinformation on health advice has highlighted the need to understand individuals’ risk perception and trust in information from different sources such as traditional media, social media, public health experts and government or related institutions during the COVID-19 pandemic (5).

Trust and credibility of information sources are considered important factors in risk communication. When an issue is new and complex, the majority lack the knowledge to directly assess the risks (6). Individuals become increasingly dependent upon information and risk assessment from experts where trust aids as a peripheral cue (6). Information presented across different media formats also varies in its quality. Traditional media typically relies on gatekeepers such as trained journalists, reporters, and editors to produce and deliver content (7). However, the emergence of online news, citizen journalism, and social media has disrupted the traditional news model. Throughout the COVID-19 pandemic, digital media played a crucial role in distributing health information, coordinating medical resources, and promoting public health campaigns. Nonetheless, digital platforms were also plagued by misinformation and conspiracy theories, undermining trust and impeding mitigation efforts (3, 8).

The COVID-19 pandemic coincided with a proliferation of sources providing health information and misinformation (9). These include alternative media platforms whose reach could cross geographical borders and social strata. This proliferation competes with institutional messaging, and research has shown that acceptance of heterodox COVID-19 narratives is associated with lack of trust in public health institutions and scientists (10). In addition, one prominent cause of resistance to public health measures which persisted throughout the COVID-19 pandemic (11), has been the lack of trust in established organizations such as the World Health Organization (WHO) (6) and traditional media organizations (12). Misinformation from credible sources compromises the efforts of public health officials in charge of administering the pandemic response efforts in the country (9).

Under these conditions, compliance with public health measures such as social distancing guidelines, movement restrictions and mask requirements has varied systematically with levels of trust in policy-makers during the COVID-19 pandemic (13, 14). Hence, higher level of trust toward certain health information sources and public trust based on perception of government competence, fairness and transparency may influence public compliance with advocated health behaviors, decreasing health risks and managing the crisis (15). For example, experiences from all over the world especially in China showed how people’s risk perception of COVID-19 could directly affect their follow-up response behaviors (16). Higher levels of risk perception were found to be associated with higher intention to engage in preventive behavior, such that if they believed that the risk of COVID-19 was high and dangerous, they would cooperate with the government’s pandemic control measures, and strictly adhere to the restrictions (16).

On the other hand, if they believed that COVID-19 was just like the influenza flu, they would ignore or delay adherence to the government’s regulation and mingle in crowds in public places which could lead to further spread of the infection and cause a wider spread of the disease (16). Hence, it has been shown that the trajectory of an infectious disease could often be determined by the behavior of individuals, and the behavior in turn is related to individual’s risk perception (17).

Other studies (18, 19) conducted during previous pandemics identified numerous psychosocial variables that potentially influenced individuals’ protective behavior. However, one factor that was found to be a crucial predictor was the level of trust in the sources of the health information (16, 20, 21). As such, public trust in the government’s competence is crucial in preventing the spread of misinformation and reducing psychological distress during the pandemic. Singapore is a small independent island situated in Southeast Asia with a multi-ethnic population of 5.6 million (74.1% Chinese, 13.6% Malay, 9.0% Indian and 3.3% others) (7). Despite being a small country, Singapore managed to contain the Covid-19 outbreak with minimum disruption to daily life largely due to their pandemic’s taskforce that was established after the SARS (severe acute respiratory syndrome) outbreak in 2003 (22). Shortly after the WHO announced the COVID-19 outbreak, Singapore’s government and related institutions acted quickly and took proactive measures such as mandatory 14-days quarantine for all residents returning from other countries, border control, contact tracing and self-isolation at home, consequently reducing community transmission (23). The present study aims to (i) explore the general population’s trust in COVID-19 information from different sources and trust in the ability of the WHO, government departments and related institutions in handling the pandemic in Singapore and (ii) explore the different factors associated with perceived risk of infection among Singapore residents.

## Methods

### Participants

A total of 1,129 participants were recruited in a cross-sectional study examining the well-being and resilience of the Singapore population (17) between May 2020 to June 2021. Inclusion criteria of the study were: (1) those who had provided consent for re-contact during Singapore Mental Health Study (SMHS) 2016; (2) Singapore citizens or Permanent Resident residing in Singapore aged 21 years and above; (3) able to speak English, Chinese, or Malay language. Exclusion criteria were: (1) uncontactable due to change in contact details; (2) those on long-term hospitalization or institutionalization throughout the study period.

### Procedure

Participants were recruited through phone calls by experienced study team members between May 2020 to June 2021. In line with physical distancing measures, participants were encouraged to participate in the interviewer-administered survey through the Zoom videoconferencing platform. Electronic informed consent was obtained via ‘Zoom’ from all participants prior to their enrolment and appropriate measures were taken to ensure confidentiality and data privacy. For those participants who preferred a face- to -face session, they were re-contacted after the Circuit Breaker period when in-person contact with participants was allowed (*n* = 122). Likewise, written informed consent was obtained from these participants prior to their enrolment. Ethics approval (DSRB 2020/00462) was obtained from the Domain Specific Review Board of the National Healthcare Group, Singapore. Clinical psychologists and psychiatrists who were part of the team followed up with participants who reported any concrete suicide plan(s) or attempt(s) in the 2 weeks prior to the interview. A comprehensive description of this study has been published in an earlier article (24).

Upon completion of the questionnaires, participants were reimbursed with SGD 40 as an inconvenience fee either through cash or cashless payment methods.

### Instruments

#### Sociodemographic questionnaire

Socio-demographic information (e.g., age, gender, ethnicity, marital status, religion, and highest education) were collected using a structured questionnaire.

#### Trust questionnaire

(i) *Trust in **COVID-19 INFORMATION** from different sources*

Participants were asked how much they trusted six sources namely traditional media, social media, governments and/or public health authorities, family doctor, local scientists, and the World. Health Organization (WHO) on COVID-19 related information. Responses were measured on a discrete scale of 1 to 10 whereby higher scores indicated higher trust (scores of 1–3 are classified as low trust, 4–7 as neutral, and 8–10 as high).

(ii) *Trust in **ABILITY** of the World Health Organization (WHO), government departments and related institutions in handling the pandemic in Singapore*

Participants were asked how much they trusted in the ability of government departments, related institutions, and the WHO, in the handling of the pandemic. Responses were measured on a discrete scale of 1 to 10 whereby higher scores indicated higher trust. The scale was locally developed by a multidisciplinary team comprising experts of the questionnaire methodology, experts in public health and preventions, and statisticians for the purpose of the study (25, 26).

#### Perceived risk of infection (self)

Participants were asked to rate their perceived risk of contracting COVID-19. This was addressed using two timeframes, where current risk was assessed by “What do you think is your current chance of getting infected with COVID-19?” and future (1 month) risk by “What do you think is your chance of getting infected with COVID-19 in the next month?” Responses were measured on a discrete scale of 1–7 whereby higher scores indicated certainty of getting infected with COVID-19 (score of 1–3 are classified as low perceived risk, 4 as neutral and 5–7 as high).

### Statistical analysis

Descriptive statistics were conducted to determine the frequencies of each of the trust levels as well as the description of the sociodemographic characteristics of the sample. Associations between sociodemographic characteristics and level of trust in COVID-19 information with perceived risk of contracting COVID-19 were examined with the use of multiple logistic regressions by which current and one-month perceived risk was the dependent variable and sociodemographic characteristics and the level of trust in COVID-19 information were the independent variables. Adjusted odds ratios (^a^OR) and 95% confidence intervals (CI) were reported to determine the associations between variables in the multivariate logistic regression model (27). Additionally, to determine if any specific demographic were more likely to trust a certain type of media, logistic regressions were run with sociodemographic characteristics as the independent variables and trust as the dependent variable. IBM SPSS Statistics software version 23 was used to run all analyses. Response rate was about 54.8%.

## Results

### Sample characteristics

Socio-demographic characteristics of the participants are shown in Table 1. A total of 1,129 participants with a mean age 42.20 (SD = 14.97) years participated in the study. The sample comprised majority of males (53.3%), Chinese (35.3%), married (60.3%), those with a tertiary education (i.e., ‘A’ Level/ITE/Diploma/ Pre-University/, Degree/Postgrad Degree and Others) (82%) and with a religion (86%).

**Table 1:** Socio-demographic characteristics of the sample.

Variables		Overall sample (*n* = 1,129)
Age		N (%)
	21–34	426 (37.7)
	35–64	580 (51.4)
	65+	123 (10.9)
Gender		
	Male	602 (53.3)
	Female	527 (46.7)
Ethnicity		
	Chinese	398 (35.3)
	Malay	263 (23.3)
	Indian	293 (26.0)
	Others	175 (15.5)
Marital status		
	Never married	363 (32.2)
	Married	681 (60.3)
	Divorced/Widowed/Separated	85 (7.5)
Religion		
	Yes	971 (86.0)
	No	158 (14.0)
Highest education		
	Secondary and below	202 (17.9)
	‘A’ Level/ITE/Diploma/Pre-university	396 (35.1)
	Degree/Postgrad Degree	481 (42.6)
	Others	50 (4.4)

### Trust in information from different sources and ability of the WHO, government departments and related institutions in handling the pandemic

The level of trust in the information from different sources and the ability of the WHO, government departments and related institutions in handling the pandemic is presented in Table 2. A majority of 85.5% reported high trust in COVID-19 information from the government and related institutes like the Ministry of Health and Multi- Ministry Taskforce (to direct the national whole-of-government response to the COVID-19 outbreak). 85.4% reported high trust in the ability of government and related institutes like Ministry of Health and the Multi- Ministry Taskforce in handling the pandemic in Singapore. Participants also reported high trust in COVID-19 information from local public health or infectious disease experts (84.4%) and traditional media (77.2%). Out of all the sources of information, social media was rated the lowest (27.0%) for trust in COVID-19 information (Table 3).

**Table 2:** Trust in COVID-19 information from different sources and the ability of the WHO, government departments and related institutions in handling the pandemic.

Trust in the sources of COVID-19 information	Low		Neutral		High	
	N	%	N	%	N	%
1. Traditional media	57	5.0	197	17.4	872	77.2
2. Social media	316	28.0	487	43.1	305	27.0
3. Local public health and/or infectious disease experts	37	3.3	139	12.3	953	84.4
4. Government departments and/or related institutes	42	3.7	120	10.6	965	85.5
5. WHO	130	11.5	290	25.7	691	61.2
Trust in the ability to handle the pandemic						
6. Ability of government departments and related institutions to handle the pandemic	32	2.8	129	11.4	964	85.4
7. Ability of WHO to handle the pandemic	196	17.4	341	30.2	572	50.7

**Table 3:** Factors associated with the perceived risk of COVID-19 infection (self).

	Current perceived risk of COVID-19 infection				One-month perceived risk of COVID-19 infection			
Variables	^a^OR	95% CI		*p*-value	^a^OR	95% CI		*p*-value
		Lower	Upper			Lower	Upper	
Age								
65+	2.05	0.63	6.69	0.23	0.77	0.14	4.36	0.77
35–64	2.70	1.26	5.80	0.01*	1.50	0.60	3.73	0.39
21–34	Ref.				Ref.			
Gender								
Female	0.68	0.39	1.21	0.19	0.59	0.29	1.21	0.15
Male	Ref.				Ref.			
Ethnicity								
Malay	1.26	0.61	2.61	0.53	1.74	0.68	4.44	0.25
Indian	0.41	0.18	0.93	0.03*	1.01	0.38	2.70	0.99
Others	0.59	0.25	1.38	0.22	0.89	0.28	2.79	0.84
Chinese	Ref.				Ref.			
Religion								
Yes	3.87	1.27	11.81	0.02*	2.03	0.61	6.71	0.25
No	Ref.				Ref.			
Education								
Secondary and below	1.31	0.57	3.00	0.52	1.53	0.52	4.55	0.44
‘A’ Level/ITE/Diploma/Pre-University	1.14	0.60	2.18	0.69	1.84	0.83	4.08	0.13
Others	2.16	0.65	7.22	0.21	2.13	0.41	11.02	0.37
Degree/Postgrad degree	Ref.				Ref.			
Marital status								
Never married	1.96	0.93	4.10	0.08	1.72	0.68	4.35	0.25
Divorced/Widowed/Separated	1.34	0.43	3.05	0.80	1.38	0.42	4.60	0.60
Married	Ref.				Ref.			
Trust in the COVID-19 information from social media								
Low	2.39	1.09	5.24	0.03*	1.07	0.38	3.01	0.89
Neutral	1.59	0.74	3.39	0.23	1.68	0.71	4.00	0.24
High	Ref.				Ref.			
Trust in the COVID-19 information from government departments and/or related institutes								
Low	0.47	0.07	3.35	0.45	0.60	0.86	4.14	0.60
Neutral	1.59	0.67	3.81	0.30	1.88	0.69	5.12	0.22
High	Ref.				Ref.			
Trust in the ability of government departments and related institution in handling the pandemic								
Low	4.64	0.80	26.88	0.09	5.75	1.02	32.45	0.05*
Neutral	1.87	0.80	4.36	0.15	2.75	1.08	7.04	0.03*
High	Ref.				Ref.			

*Bold values indicate significant *p* value *p* < 0.05. ^a^OR, adjusted odds ratio; 95% CI, 95% confidence interval; Ref., reference categories. The Nagalkerke R-Square value for current perceived risk (0.105) and one-month perceived risk (0.114) showed that the model explains 10.5 and 11.4% of the variation of perceived risk of COVID-19 infection, respectively. The Omnibus tests of model coefficients give a chi-square of 91.063 (*p* < 0.001) for current perceived risk and 91.522 (*p* < 0.001) for one-month perceived risk indicating good fit for both models. The analysis estimated the overall accuracy of 64.1% for current perceived risk and 72.6% for one-month perceived risk in correct prediction of the probabilities.

### Correlates of perceived risk of COVID-19 infection (to self)

Participants aged 35–64 (vs. 21–34) years were significantly associated with high current perceived risk of infection (^a^OR: 2.70, 95% CI 1.26–5.80). Indians (versus Chinese) were significantly associated with low current perceived risk of infection (^a^OR: 0.41, 95% CI 0.18–0.93). Those with a religious affiliation (vs. those without) were significantly associated with high current perceived risk of infection (^a^OR: 3.87, 95 CI 1.27–11.81). Low trust in social media (versus high trust) was associated with high current perceived risk of infection (^a^OR: 2.39, 95% CI 1.09–5.24). As for the future (one-month) perceived risk of COVID-19 infection, participants with low trust in the ability of government departments and related institutions in handling the pandemic were associated with a high future (one-month) perceived risk of infection (^a^OR: 5.75, 95% CI 1.02–32.45) (Table 3).

## Discussion

Our research investigated the association between the public’s trust in COVID-19 information from different sources and the ability of the WHO, other government departments and related institutions in handling the pandemic in Singapore. Along with this, the current research investigated the factors associated with perceived risk of infection among Singapore residents. The study found that majority of the residents reported high trust in the government and related institutions’ ability in handling the pandemic in Singapore. As mentioned previously in the literature review, perception of government competence and transparency may possibly influence management of the crisis (15). For example, as COVID-19 began to unfold in Singapore, key government officials openly addressed the scientific uncertainties around the virus, the search for a vaccine and detailed summary of contact tracing (4). In addition, frequent press conferences held by the Prime Minister speaking in the language of the targeted audience without the use of translators was key in building trust and promoting affective beliefs about the institutional behavior and competence especially in a multi-ethnic population like Singapore (4). As such, with all the meticulous contact tracing procedures and information communicated heavily through credible sources, it established a positive perception of the government’s risk management and high level of trust in the Singapore government.

The findings of the current study also support the notion that those with a lower trust in the government’s ability was associated with a higher level of perceived risk of infection. A local study (4), showed that those who had a very positive perception of the government’s risk management and communication efforts, expressed a very high level of confidence in government and healthcare system. In turn, they considered their risk of infection to be very low because they felt that the government had been transparent, highly competent, and effective. Whereas those who were more skeptical and found the government’s approach to be confusing expressed higher level of anxiety and risk of infection.

We also established that the older participants in our sample had a higher current perceived risk of COVID-19 infection. We believe that objective information on infection rates might have misled younger individuals to be influenced by ageism and biased risk (28). Hence, given the high engagement of youth with multiple information sources such as medical sources and mass media coverage, younger individuals may have gathered the message that COVID-19 infection mostly concerns older individuals who are at higher risk of being infected or dying (29).

This study makes another interesting contribution to the literature. The results show that those with lower level of trust in social media was associated with higher current perceived risk of infection. During the early stages of an infectious diseases outbreak, traditional media platforms such as television news, newspaper, radio stations, governments and/or public health officials or institutions may not always provide the public sufficient information in a timely manner, due to the lack of disease-related information (30, 31). Hence, individuals may turn to social media platforms particularly Facebook, Instagram, Youtube or Twitter feeds as an effective and immediate information tool to communicate relevant information to others (28). Unsurprisingly, these sources are unverified and therefore, foster the spread of conspiracy theories, myths, and false information which can consequently fuel uncertainty and psychological problems for instance, fear and anxiety in the population (16, 25, 28). However, misinformation surrounding COVID-19 also involved downplaying the seriousness of the pandemic with several influential accounts stating that COVID-19 was not more severe than the ordinary flu and greatly discouraged the use of face masks and other preventive measures (32, 33). It is possible that the people who chose not to believe in such information, feared the virus and its impact on their wellbeing, and therefore, they may have perceived a higher degree of risk that they will be infected. Further research is needed to evaluate the association between risk perception and the use of social media platforms.

Interestingly, the study revealed that those of Indian ethnicity reported lower current perceived risk of infection as compared to Chinese ethnicity. One possible explanation could be related to beliefs regarding differences in COVID-19 infection rates across countries contributed by media and symptom reporting in hospitals. China was the first country to experience the initial phase of COVID-19 pandemic outbreak and adopted different safety measurements in different stages (2). During the initial phase of the pandemic outbreak in early 2020, China had higher COVID-19 infections rates and deaths as compared to India (2). However, though China’s daily cases were high, it was mainly concentrated in the early phase; China quickly adopted active screening, tracking of cases and strict city closure measures which slowed the increase of COVID-19 cases (2). In contrast, daily infection cases were low in India during the early phase of the pandemic, however it continuously rose during the later stages (2). Thus, the media reporting and the study recruitment being in the early phase of the pandemic, could have resulted in biased risk perception among these ethnic groups in Singapore. However, further research is needed to better understand these ethnic differences in perceived risk of infection.

### Limitations

There are some limitations in this study. Firstly, collection of data through videoconferencing was encouraged. A significant number of older adults were reluctant to participate as many were not comfortable with using and signing the consent form through the online platform. Secondly, the pandemic evolved through many stages corresponding to changes in health measures, infection rates and restrictions, and thus trust levels may have varied in the population. Thirdly, the validity and reliability of the questionnaires content used in the study were not formally assessed. Lastly, data collected for this study comprised a small sample size which may not be sufficiently representative of the population.

## Conclusion

In summary, effective public health messaging during a pandemic is crucial as it influences public compliance, advocates health behaviors, decreases health risks and helps in the management of the crisis. The present study provides important insights into the level of trust on COVID-19 information from different sources, and the ability of the government and related institutions in handling the pandemic in Singapore. Future qualitative studies are recommended to facilitate better understanding of public trust and identify strategies on how it can be further strengthened in preparation for future public health responses to crises.

## Data availability statement

The raw data supporting the conclusions of this article will be made available by the authors, upon reasonable request.

## Ethics statement

The studies involving humans were approved by Domain Specific Review Board of the National Healthcare Group, Singapore (DSRB 2020/00462). The studies were conducted in accordance with the local legislation and institutional requirements. The participants provided their written informed consent to participate in this study.

## Author contributions

FD: Data curation, Investigation, Methodology, Project administration, Writing – original draft, Writing – review & editing. BT: Formal analysis, Software, Writing – review & editing. SaS: Conceptualization, Data curation, Investigation, Methodology, Project administration, Supervision, Writing – review & editing. YZ: Data curation, Investigation, Methodology, Project administration, Writing – review & editing. ShS: Conceptualization, Data curation, Investigation, Methodology, Writing – review & editing. PS: Conceptualization, Data curation, Investigation, Methodology, Writing – review & editing. SC: Funding acquisition, Supervision, Writing – review & editing. MS: Funding acquisition, Investigation, Project administration, Resources, Supervision, Validation, Visualization, Writing – review & editing.
